# Awareness and experiences on core outcome set development and use amongst stakeholders from low- and middle- income countries: An online survey

**DOI:** 10.1371/journal.pgph.0002574

**Published:** 2023-12-05

**Authors:** Jamlick Karumbi, Sarah Gorst, David Gathara, Bridget Young, Paula Williamson

**Affiliations:** 1 Department of Health Data Science, University of Liverpool, Liverpool, United Kingdom; 2 Health Systems Research, KEMRI-Wellcome Trust Research Programme, Mvita, Kenya; 3 Centre for Maternal, Adolescent, Reproductive & Child Health (MARCH), London School of Hygiene and Tropical Medicine, London, United Kingdom; 4 Department of Public Health, Policy and Systems, University of Liverpool, Liverpool, United Kingdom; PLOS: Public Library of Science, UNITED STATES

## Abstract

Harmonization of outcomes to be measured in clinical trials can reduce research waste and enhance research translation. One of the ways to standardize measurement is through development and use of core outcome sets (COS). There is limited involvement of low- and middle-income country (LMIC) stakeholders in COS development and use. This study explores the level of awareness and experiences of LMIC stakeholders in the development and use of COS. We conducted an online survey of LMIC stakeholders. Three existing COS (pre-eclampsia, COVID-19, palliative care) were presented as case scenarios, and respondents asked to state (with reason(s)) if they would or would not use the COS if they were working in that area. Quantitative data were analyzed descriptively while qualitative data were analyzed thematically. Of 81 respondents, 26 had COS experience, 9 of whom had been involved in COS development. Personal research interests and prevalence of disease are key drivers for initiation/participation in a given COS project. Most respondents would use the COS for pre-eclampsia (18/26) and COVID-19 (19/26) since the development process included key stakeholders. More than half of the respondents were not sure or would not use the palliative care COS as they felt stakeholder engagement was limited and it was developed for a different resource setting. Respondents reported that use of COS can be limited by (i) feasibility of measuring the outcomes in the COS, (ii) knowledge on the usefulness and availability of COS and (iii) lack of wide stakeholder engagement in the COS development process including having patients and carers in the development process. To ensure the development and use of COS in LMICs, collaborations are essential in awareness raising on COS utility, training, and COS development. The COS also needs to be made accessible in locally understandable languages and feasible to measure in LMICs.

## Introduction

The practice of evidence informed health care has been ongoing for the last few decades [1]. A key principle of this practice is collation of research findings in a systematic way. For collation of findings to be sensible and inform policy and practice, there is need to choose the right outcomes. One way of determining the ‘right’ outcomes and how to measure them is through development of core outcome sets (COS). COS are agreed-on minimum standardized outcome sets that should be measured and reported in all research in a given health area [2]. Development and use of COS has the potential of improving reporting of patient-relevant outcomes and enables pooling and comparison of findings. This would reduce research waste by enhancing comparability of results and hence improving research translation and use [3,4].

COS have been developed for various disease areas [5]. Statistics from the Core Outcome Measures in Effectiveness Trials (COMET) Initiative data base (www.comet-initiative.org), to-date, show there are over 800 (440 published and >400 ongoing) COS across various health areas [6].

Even though there has been progress in including low- and middle-income countries (LMIC) stakeholders in the development of COS, most COS are still being led from high income countries. By the end of 2020, only four COS had been initiated from LMICs [7].

The COS that are already developed are therefore likely to be shaped by perspectives of those from high-income countries (HICs) which may be different from LMICs.

### Why do we need to include LMIC stakeholders in COS development?

The limited engagement of LMIC stakeholders in the COS development process may limit COS use and lead to research waste that the COS was intending to minimize. Even though, some research outcomes may be applicable to both LMICs and HICs, there could be important differences in disease patterns, capabilities of the healthcare system, and priorities not only for the researchers and clinicians, but also for patients and caregivers [8,9].

### What influences inclusion of LMIC stakeholders in COS development?

Choosing appropriate outcomes to measure has been reported as priority for methodological research by LMIC researchers [10]. Despite this being a priority, there are few COS that have had LMIC stakeholder engagement. We have previously undertaken work to understand the experiences of including LMIC stakeholders in COS development by researchers from high income countries [7]. In that study, we report that where LMIC stakeholders were included in the COS development process, they were more likely to be involved in determining ‘what to measure’ than being involved in whole process. The existence of working collaborations with LMIC stakeholders was the main enabler for participation in COS development. COS developers also felt that translation of the Delphi into languages other than English, though costly, may be useful to enhance wider stakeholder participation. Some of the challenges reported by COS developers in including LMIC stakeholders in COS development were lack of adequate resources to support their involvement, and lack of networks and contacts which limited fuller participation within LMICs.

For more COS to be developed and used in LMICs, there is a need to first understand the experiences of stakeholders from LMICs in COS development and use. Knowledge of their experiences will help in designing strategies that can be implemented to enhance COS development and use in LMICs. We therefore conducted a survey of LMIC stakeholders to explore their awareness of and experiences in the development and use of COS.

## Methods

### Design

This was a cross-sectional online survey which was conducted in English and included some brief demographic questions, followed by a brief description of COS and a question on whether respondents were familiar with COS. The study report is informed by the Checklist for Reporting of Survey Studies (CROSS) [11].

### Data collection methods

The demographics and basic information items were based on guidance provided by TGHN (Based on standard items that are assessed within the network when they undertake an online survey). The technical items of the survey were generated based on literature review on LMIC stakeholder involvement in COS work [7] and was also informed by work undertaken to understand the facilitators and barriers to use of COS by clinical trialists in HIC [12] and barriers and facilitators to uptake of use of clinical guidelines in LMICs [13]. The survey was piloted amongst the survey authors and refined further before dissemination.

**Fig 1:**
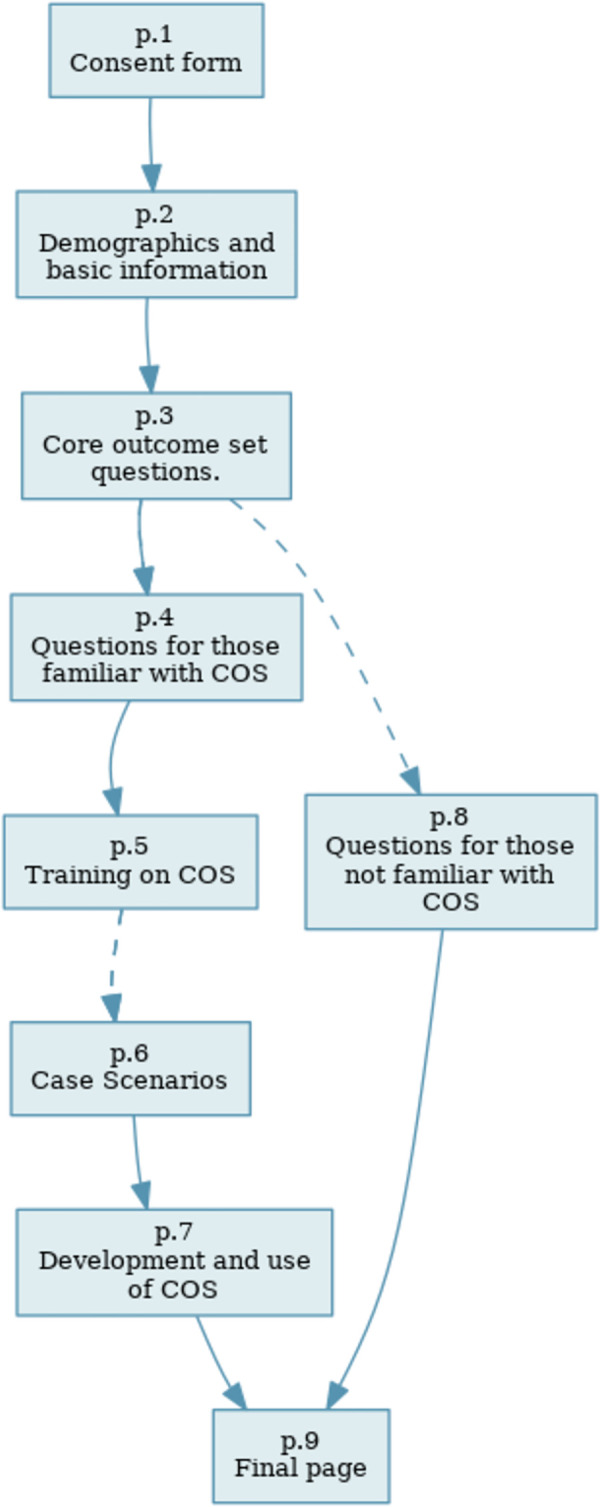
Survey map.

The survey questionnaire, which had skip logics, had a total of 23 questions divided into 8 sections. Those familiar with COS (based on the question on section 3), had 18 questions while those with no prior COS familiarity had 11 questions as shown in Fig 1.

For those familiar with COS, a series of questions on their experience with COS followed. We presented three existing COS (pre-eclampsia, COVID-19, and palliative care) as case scenarios where the respondents were asked to state (with reason(s)) if they would or would not use the COS if they were working in that area. LMIC stakeholders had been included in the development of each COS. The three COS were purposely selected to present different methodological aspects: (i) pre-eclampsia, online Delphi which included LMIC participants from 25 countries, but the consensus meeting did not involve any LMIC stakeholders [14]; (ii) COVID-19, online Delphi (survey conducted in five languages—English, Chinese, Italian, Portuguese, and Spanish), four online consensus meetings conducted in English with LMIC stakeholders from 2 countries [15,16]; (iii) palliative care, developed through and international expert consensus workshop using nominal group technique. The workshop was convened within the 9th World Research Congress of the European Association for Palliative Care in 2016. The workshop was a closed session lasting approximately 90 min with participants being divided into groups of 6 to 10 people. Stakeholders from one LMIC were involved in COS development [17].

For all respondents (those familiar and those not familiar with COS) a series of questions asking them to rate the importance of a few potential barriers and facilitators in using COS were asked. These barriers were identified not only through the review of literature on barriers and facilitators on use of COS amongst clinical trialists as described by Hughes [12] but also adapted from general barriers and facilitators that affect implementation of clinical practice guidelines [13]. They were then rated on a scale of 1 (least important) to 9 (most important). The lead author (JK) designed the questionnaire with a mix of closed and open-ended questions and the author team helped to refine it. We piloted the questionnaire with the other author team members before finalizing it.

We included a photo of the survey author in the body of the questionnaire to enhance response as suggested by Edwards et al [18]. The opening part of the questionnaire had a consenting question. For those who did not provide consent, the survey terminated at that stage. We collected data between December 2021 and February 2022 using the JISC online surveys (https://www.onlinesurveys.ac.uk/) platform. See S1 File for survey questions.

### Participant selection and recruitment

The survey targeted LMIC stakeholders who are members of The Global Health Network (TGHN). TGHN is a global open community of practice for health workers, research teams, and research organizations. It facilitates, supports, and enables research in diseases, places, and settings where there are evidence gaps. It works by sharing research methods, know-how, and data between organizations, projects, regions, and roles, and delivers capacity building, support, and training to research teams and frontline health workers (https://hub.tghn.org).

The online survey was shared to all members of the TGHN website where an invitation to participate in the survey was sent through an advertisement in TGHN monthly newsletter for December 2021. In January 2022, a website page was set up on the TGHN website for the survey and a reminder email with the link to the page sent two weeks before the closure of the survey. The email reminder was sent to members of methodology hubs within TGHN. The methods hub has approximately 12,000 members and 40% of the visits to the website are by stakeholders from researchers in LMICs, [TGHN Coordinator personal communication]. We therefore expected the email to have a reach of about 4,800 stakeholders. These respondents could either be researchers, clinicians, or patients/members of the public who are registered on the website.

### Ethics approval and consent to participate

Ethical approval was granted by the Health and Life Sciences Research Ethics Committee (Human participants, tissues, and databases) at the University of Liverpool on 19th November 2020 (reference 7661). All survey participants were provided with participant information as an attachment to the email inviting them to participate in the survey.

The consenting process was ‘written’ through provision of a response (yes/no) to the opening statement of the questionnaire “I consent to participate in the survey (yes/no).

Participants were required to respond by ticking a yes box if they consented to participate in the survey. The survey terminated immediately for those who did not consent (those who ticked no).

### Analysis of survey responses

Quantitative data from the closed questions were analyzed using simple descriptive statistics. For the questions on rating the barriers and facilitators, a range, median and interquartile range is presented from the most important to least important factor based on the median.

Qualitative data were comprised of responses to the open-ended questions and the case scenario questions where respondents were to provide an explanation on the choice they made (whether they would/ would not use the COS described in the scenario). Thematic analysis of these qualitative data was deductive, whereby the themes were pre-determined by the questions in the survey and informed by findings from an earlier survey that targeted COS developers who had included LMIC stakeholders in the process. (Manuscript in preparation). All free text was extracted on to a word document which JK read multiple times to organize the text into some initial themes and the author team reviewed the data and its assignment to the themes identified.

Excerpts from respondents’ free text responses to open-ended questions are presented accompanied with their unique identification numbers R1 to R26 and accompanied by the region that the respondent was from e.g., R2 from Africa would mean respondent number 2 from the African region. This labeling allowed us to track responses from one respondent across various questions. To help maintain anonymity, small sections of text were omitted, and these are presented as square brackets (xxx).

All authors provided their perspectives on the analysis and reporting of the data, this helped reduce domination of a single perspective on the analysis and discussions.

## Results

### General descriptions

The first page (Survey information and consenting page) was accessed 2796 times. There were 89 responses 81 of whom completed the survey and 8 did not consent to the survey and were hence screened out of the survey.

Out of 81 respondents, 26 (32%) had experience with COS. The majority were male and those who had experience in using COS had spent a slightly longer duration in their areas of practice. Most were researchers and academics and were from Africa, although there were 7 respondents who were not residing in LMICs at the time of the survey, Table 1.

**Table 1:** General descriptions of respondents.

Description	Category	Experience with COS (n = 26)	No experience with COS (n = 55)	N = 81 (%)
Gender	Male	14 (54)	32 (58)	46 (57)
	Female	12 (46)	23 (42)	35 (43)
Time spent in the main profession	median years (IQR)	11 (6–25)	6 (3–14)	8 (4–17)
Area of specialty	Nursing	3 (12)	8 (15)	11 (14)
	Public Health/Epidemiology	5 (19)	6 (11)	11 (14)
	Infectious diseases	3 (12)	5 (9)	8 (10)
	General Medicine	2 (8)	5 (9)	7 (9)
	Pharmacy	2 (8)	4 (7)	6 (7)
	Social Sciences	0	5 (9)	5 (6)
	Others^$^	11 (42)	22 (40)	33 (41)
Area of practice	Health Care Practitioner	9 (35)	17 (31)	26 (32)
	Researcher/Academic	11 (42)	27 (49)	38 (47)
	Other^£^	6 (23)	11 (20)	17 (21)
Region of LMIC^	South and Central America	3 (12)	4 (7)	7 (9)
	Asia	6 (23)	5 (9)	11 (14)
	Africa	12 (46)	44 (80)	56 (69)
	Other	5 (19) ^#^	2 (4) ^##^	7 (9)

^$^This includes categories with a frequency of less than or equal to 3 like, child health, obstetrics etc.

^£^This includes those working as independent consultants or are self-employed or in NGOs.

^See S2 File for the full list of countries of all participants.

^#^Spain (1), UK (2), Canada (2)

^##^Belgium (1), Saudi Arabia (1).

### Responses by those familiar with COS

In this section, we provide a description of the responses of those familiar with COS.

Just over a third 10/26 (38%) had COS introduced to them by colleagues or by their professional associations. Whereas most 17/26 (65%) reported having used a COS in their work, only 8/26 (31%) have initiated COS development with 2 of them being current residents of high-income countries. A literature search was the commonest source of COS information. The most reported reason for initiating COS development or being part of the development team was disease prevalence in LMIC and personal research/clinical interest. Half of the respondents had had COS awareness raising sessions whilst three had received training, all undertaken as part of general research methods training, Table 2.

**Table 2:** Characteristics of those familiar with COS.

Description	Category	n (%)
Familiarity with COS (n = 26)^	Read about COS in a trial	15 (58)
	Read about COS reported in a COS development paper	13 (50)
	Conference presentation on COS	8 (31)
	Professional colleagues/associations	10 (38)
Usage of COS (n = 26)	Used COS in my work	17 (65)
	Initiated development of COS	8 (31)
	Part of COS development team	6 (23)
Used COS (n = 17)	Africa	8 (47)
	South and Central America	2 (12)
	Asia	4 (24)
	UK and Canada	3 (18)
Area of specialty (n = 17)	Nursing	1 (6)
	Public Health/Epidemiology	4 (24)
	Infectious diseases	2 (12)
	General Medicine	1 (6)
	Data management	3 (18)
	Pharmacy	1 (6)
	Others^$^	5 (29)
Source of COS information (n = 17)	COMET database	4 (24)
	Literature review	11 (65)
	Previous experience in COS use	8 (47)
	Other**	3 (18)
Initiation of COS (n = 8)	Africa	3 (38)
	Asia	2 (25)
	UK and Canada	2 (25)
	South and Central America	1 (12)
Part of COS development team (n = 6)	Africa	3 (50)
	Asia	1 (17)
	UK and Canada	2 (33)
Motivation for involvement in COS (n = 9) (those initiating and involved in COS development)	Prevalence of disease in my country	6 (67)
	Prevalence of disease in LMICs	8 (89)
	Working Collaborations with HIC colleagues undertaking COS work	5 (56)
	Personal clinical/research interest	7 (78)
Stage of COS involvement (n = 9)	Determining the scope of the COS, i.e., as part of the research team	8 (89)
	Development of the protocol for the development of the COS–the ‘what’ to measure, i.e., as part of the research team	5 (56)
	Determining ‘what to measure’, i.e., giving their views on what to measure	9 (100)
	Determining ‘how to measure’ the COS, i.e., giving their views on how to measure	7 (78)
Training on COS (n = 26)	Yes	3 (12)
Source of training (n = 3)^^	As part of general research methods training	2 (67)
	As part of master’s degree training	1 (33)
Sensitization on COS (n = 26)	Yes	13 (50)
Source of sensitization (n = 13)	As part of general research methods training	7 (54)
	As part of conferences/webinars organized by The Global Health Network/COMET Initiative	4 (31)
	Continuous medical education session through my professional association	5 (38)
	Other^$ $^	2 (15)
Need for translation to non-English language to enhance use of COS	Yes	21 (80)
	No	3 (12)
	Not sure	2 (8)

^$^This includes categories with a frequency of 1(General surgery, Clinical trialist, Obstetrics, Child Health, Orthopedics).

*This includes respondents who indicated they were working in UK (3), Spain (1), Canada (1), Latvia (1), Belgium (1).

**From colleagues, university research work, US NIH website.

^^ This are part of the team that have been involved in initiation of COS development.

^$ $^From ERC and online.

### Case scenarios

Most of the respondents (69%) stated that they would use the pre-eclampsia COS, 73% stated they would use the COVID-19 COS, but only 46% stated that they would use the palliative care COS as shown in Table 3.

**Table 3:** Use of COS from three case scenarios.

COS	Category	N = 26 (%)
Pre-eclampsia (online Delphi with a physical consensus building)	Yes	18 (69)
	No	2 (8)
	Not sure	6 (23)
COVID-19 (online surveys with translations into 5 languages and online consensus building)	Yes	19 (73)
	No	2 (8)
	Not sure	5 (19)
Palliative care (developed during an international conference)	Yes	12 (46)
	No	5 (19)
	Not sure	9 (35)

We analyzed the reasons provided by respondents as to whether they would or would not use the COS described in the case scenarios. Eight respondents provided general answers, while the remaining eighteen respondents provided specific answers as to why they would or would not use a given COS as shown in S3 File. We highlight six themes that we identified from both general and specific responses. The first theme relates to the general answers while the other five (II to IV) were identified from the specific answers.

### I. Enhance comparability of research findings

Respondents stated that they were likely to use a COS to enhance standardization of outcomes and hence comparability of their research findings.

“To make sure that my results could be comparable to other studies” (R4 from Africa)

“the COS would serve as the basis and standardized mold for guiding the researchers.” (R5 from Africa)

### II. Level of stakeholder engagement

The level of stakeholder engagement was reported by respondents as influencing whether they would use a COS or not. When there was wide stakeholder engagement, respondents reported that they were likely to use the COS even though it was developed in a different setting.

“Because the process through which the COS were developed involved almost all the necessary stakeholders on the topic or issue. Though, its limitation is that it was only conducted in English language” (R25 from Africa).

On the other hand, when respondents perceived that only a small group of stakeholders were involved, then they were unlikely to use the COS.

“it is a consensus only from a small group from different countries, in my point of view it is not reliable and could not generalized” (R26 from South and Central Americas)

### III. Settings in which a COS was developed

Some of the respondents felt that when a COS is developed in a setting (different social economic levels and healthcare system development levels) that is different to their own then it may not be easily transferable into their setting. For example, COS developed in HICs may not be easily transferable to LMICs.

“These COS may not be relevant to me because the responses were mostly from High income countries” (R9 from Asia)

“All researchers leading the team are (…) (a country in Europe) residents meaning they might only have a developed country view of the study. And being the technical team, it will automatically influence the end results which may not reflect the real outcome because they might ignore some key variables or timing or procedure that might have been added by someone who live in LMIC” (R22 from Africa)

### IV. Feasibility of measuring the outcomes

One of the considerations that respondents reported was feasibility of measuring the outcomes in different settings that have been agreed upon. This could be due to lack infrastructure for measuring the outcomes or when the number of outcomes suggested is too big.

“22 COS in total, with no hierarchy or grouping according to objectives or research phases, seems too much to me, and may bring confusion” (R20 from South and Central America)

“Liver enzymes, renal function and platelets not easily measured in my setting. Expertise to identify retinal detachment not available. Expertise to recognize cortical blindness limited. Neonatal seizures would only be recognized if clinical. Neonatal EEG (electroencephalogram) not routinely available. Intubation not available at all study sites.” (R23 from Africa)

### V. Comprehensiveness of COS

Despite noting the importance of the outcomes in a COS being feasible and not too numerous to measure, respondents reported that they were likely to use a COS if it had covered all the relevant outcomes in their area of work. This seems to contradict the response of lack of feasibility due to the number of outcomes being too numerous without a clear guidance on which outcomes to choose given circumstance. It may be an indication that COS developers need to strike a balance between comprehensiveness and feasibility of the outcomes in a COS.

“I would consider including economic, job, education and daily life impact of COVID-19 diagnosis or suspected infection. Despite being only 2 low- and middle-income countries in the study, the wide range of variability in social and cultural environments from these countries could allow a better understanding regarding the role of COVID-19 in health of people” (R12 from South and Central Americas)

“This covers the major critical events with COVID hence valid to be used as Core outcomes” (R19 from Asia)

### VI. Language used during the development of the COS

Most respondents indicated that where multiple languages were used during the development process a COS is more likely to be used than when its development was conducted in English language only.

“The COS were also generated through involvement of all the necessary stakeholders. Additionally, the process used five languages and more diverse stakeholders. Hence, it’s more detailed or thorough that the first process” (R25 from Africa)

“because the data are from different countries and languages, what reduce de bias and the literature evidence was from clinical trials. In addition, the sample was considerable good number, in my opinion, the patient sample could be larger.” (R26 from South and Central Americas)

### Facilitators and barriers to use of COS

For this section, we asked all the respondents (N = 81) how they would rate a set of pre-identified facilitators and barriers to using COS on a scale of 1 to 9 with 1 being least important and 9 being most important. We have used the median score, followed by the Inter Quartile Range (IQR), to order the factors as shown in Table 4.

**Table 4:** Rating of motivators and barriers for COS use on a scale of 1 least important to 9 most important.

Factor	Range	Median (IQR)
**Motivators**		
Availability of validated tools to measure the outcomes that have been proposed in the given COS	5–9	9 (8–9)
Knowledge of the usefulness of COS in research and clinical practice	5–9	9 (7–9)
Outcomes in a COS will be more patient-centered due to stakeholder involvement and participation	5–9	8 (8–9)
Having stakeholders from settings similar to mine (geographical and/or resource setting) being involved in the COS development	1–9	8 (7–9)
Endorsement by my professional network/association	2–9	7 (6–8)
Recommendations by funders or regulatory agencies in my country of work	2–9	7 (6–9)
**Barriers**		
Lack of knowledge about existence of COS in my area of work	2–9	9 (6–9)
Lack of validated tools to measure the outcomes that are outlined in the COS	1–9	7 (5–9)
Outdated COS measurement methods	1–9	7 (5–8)
Increasing burden to the clinicians and patients when additional outcomes need to be reported	1–9	7 (5–8)
Lack of skills on how to apply COS	1–9	7 (4–9)
The need for me to use my own outcomes or locally contextualized outcomes	1–9	7 (4–8)
Inapplicability of COS developed in other geographical and resource settings to my own setting	1–9	7 (4–8)
Lack of COS material in my local language	1–9	6 (5–9)
Outdated COS in my line of work	1–9	6 (5–7)
Too wide or narrow scope of COS which have already been developed	1–9	6 (4–8)
Costly methods of measuring the outcomes in the COS	1–9	6 (3–8)
COS would limit the range of outcomes I would like to assess or track	1–8	5 (3–7)

Most respondents rated the availability of validated tools to measure the proposed outcomes as the most important facilitator, while recommendations by professional associations and research funding agencies were rated as being least important. They rated lack of knowledge on COS, measurement of the outcomes and lack of tools and/or outdated outcome measurement tools as the most important barrier. The least important barrier was that a COS might limit the range of outcomes that would be measured in a given research area.

### Strategies to improve LMIC stakeholder engagement

We sought to obtain views from all respondents (N = 81) on what they thought would be useful strategies to widen stakeholder engagement in COS development and use. Response data are given in S4 File. We have categorized the responses into four thematic areas.

### I. Enhancing partnership and collaborations

Respondents suggested that enhancing partnerships and working collaborations with those already undertaking COS work would enable LMIC stakeholders to be part of the process.

“The application of partnership and collaboration principles” (R3 from Africa)

“Involvement of institutions especially universities LMICs during the early phase and training of man powers in these marginalised territories.” (R14 from Africa)

### II. Sensitization and training and on COS

Respondents indicated that having more sensitizations on what COS is and the usefulness of using COS and provision of trainings could improve LMIC engagement. These trainings could be done through academic institutions as part of the training curriculum and/or as part of on job trainings through professional associations and other relevant government agencies like regulators, funders, guideline developers etc.

“Hardly anyone in my circle has heard of COS. The first step would be to disseminate knowledge on what COS is and what are its benefits followed by training sessions -online or offline” (R16 from Asia)

“(1) Increased awareness about the existence of COS and the need to use it. This can be done through engagement with various professional bodies and ministries of health in these regions (2) Involvement of governments in the regions, to "buy into” the need for COS, with a view to ensuring political will and policies to support development of COS for treatment of each clinical condition, and ensure it’s used. (3) Research funders should also recommend the use of COS for any clinical trial they will be sponsoring in the regions (4) Regular training of researchers on COS (5) International or foreign researchers (for high-income countries) should recommend/suggest the need to have and use COS to their collaborators in LMICs” (R25 from Africa)

### III. Enhance availability of COS

Respondents felt that COS that have already been developed need to be made available in a way that is accessible to them, this could include having them published in local languages.

“Advocacy and sensitization, involvement of the stakeholder while developing the COS. Also, contextualizing the COS and having it in indigenous languages is imperative. I have taken the WHO Mass Online Open Course in Implementation Research, such training can be contextualized for stakeholders and end-users of the COS to enhance acceptance, applicability, and fidelity.” (R44 from Africa)

### IV. Continuous monitoring and evaluation of COS implementation

Respondents indicated that for implementation of COS to be effective, regular monitoring of the data collected on the core outcomes is essential. This provides not only an avenue for providing feedback loops to the clinicians, researchers, and COS developer but also helps create interest in COS.

“Requesting for feedback from healthcare professionals in practice wherever possible and giving them a chance to collect the opinions of the patients they service will increase interest and build capacity” (R13 from Africa)

“…. Routine monitoring and evaluation measures should also put in place for smooth implementation in general”. (R57 from Africa).

## Discussion

In this study, we have explored the level of awareness about COS amongst LMIC stakeholders. We have also explored the experiences of those who are familiar with COS and what they think can help improve development and use of COS in LMICs.

Seventeen out of eighty-one respondents had used a COS in their work with almost half of them being from the African continent. LMIC stakeholders were mainly involved in the ‘what to measure’ stage and this was also evident from an earlier survey of COS developers who were predominantly from HICs (Manuscript in preparation). Inclusion of LMIC stakeholders in the whole process of COS development has been suggested by both COS developers and LMIC stakeholders as one of the strategies that would see more COS use in LMICs. Additionally, since only a small proportion of COS are initiated from or involve stakeholders from LMICs [7], where COS are already developed for a given area, perhaps a process of validation of the COS in LMIC settings could be useful in assessing if the COS is relevant to LMIC contexts (and hence use) and for the various groups like patients, clinicians and researchers who may not have been involved in the COS development process or were only involved in the later stages of COS development.

Like the findings of our survey on COS developers who had included LMIC stakeholders in COS development, personal clinical interest, and prevalence of a disease in LMICs were key drivers of involvement in COS development (Manuscript in preparation). Perhaps entities like COMET Initiative, could flag areas with greatest need for COS to encourage people/groups to address e.g., through collaborating with research priority setting groups in LMICs which could provide an entry point for COS in the prioritized areas.

From the case scenarios, three factors seem critical in influencing the decision to use (or not) existing COS. Firstly, the range and number of stakeholders engaged in developing the COS. For example, where a wide range of stakeholders from different settings are involved i.e., health professionals, public participants and patients with lived experiences, respondents indicated they were likely to use the COS. This is probably because all relevant outcomes are likely to be part of the COS if a diversity of stakeholders have been included in its development. Hughes et al reported similar findings where lack of engagement of the end users of COS and those with lived experiences in a given diseases were reported to lead to lower COS uptake [12]. Additionally, engagement of the health workers responsible for routine data capture in the COS development process can help in facilitating the integration of outcomes in routine data collection systems, hence enhancing their use.

Secondly, the feasibility of measuring the outcomes in the LMIC setting is a major determinant of COS uptake. A COS is unlikely to be used when there is a challenge in measuring the outcomes, for example some of the outcomes in the pre-eclampsia COS were reported as not being ‘measurable’ in low resource settings. These could be due to lack of guidance on how to measure the outcome, lack of measuring equipment/tools, lack of expertise in measuring the outcome in clinical practice and sometimes the outcomes being too many to measure. It is therefore useful to ensure that as developers aim for the COS to be as comprehensive as possible, the number of outcomes need to be realistic or a guidance for circumstances when to use given outcomes be provided. For example, Webbe et al developed a COS for neonatal care with a total of 12 outcomes but gave guidance that two of the outcomes to be used only when dealing with preterm babies [19].

Lastly, respondents were clear that the resource setting in which the COS was developed would impact on their COS use. This could be due to two issues: (i) feasibility of outcome measurement and documentation during research or in routine clinical practice. This is mostly linked to the capability of the health system. Resource limited settings have systems that may not have the ability to measure, document the outcomes that have been agreed on; (ii) a varying disease burden and epidemiology coupled with a differing level of patient and public partners being involved in research in HICs compared LMICs could mean that the prioritized outcomes that end up in the COS might differ. For example, in the COS for neonatal research, brain injury on imaging was prioritized by public partners in HIC, but these might not be prioritized in LMIC as brain imaging is not a routine diagnostic test in such settings [19].

Lack of translation was not ranked as the most important barrier although it was noted that it could be a barrier in situations where the COS has patient and public partners who may not be conversant with the English language. This reflects findings from our COS developers survey where respondents indicated that they did not consider translation since the majority of LMIC stakeholders were clinicians and researchers who were conversant with the English language. (Manuscript in preparation). Even though there is paucity of evidence on whether translation of the Delphi study improves uptake of the COS, where translation has been undertaken, there was no significant increase in the number of participants in the Delphi process [20]. This could be a pointer that translation on its own without wider stakeholder engagement efforts may not bear the envisioned benefits.

Lack of knowledge of the existence of COS for a given research area was reported as a key hindrance to use of COS. This was also reported by COS developers from HICs. It is therefore not surprising that it was ranked highly as a barrier to use of COS and as such, sensitization and training on COS was a recurring theme in the plausible solutions to improve the use of COS. This training could be undertaken in various settings and leverage on existing structures such as professional associations, higher education institutions and online webinars by COS developers and trial methodologists. The sensitization and training should be coupled with ensuring there is access to COS resources through proper dissemination of the COS [21].

Even though COS developers cited lack of funding as one of the key barriers to including LMIC stakeholders in COS development, this was not directly cited by LMIC stakeholders. It is, however, a likely key challenge since the suggested solutions are heavy on awareness raising and training on COS, translation of study materials into local languages and engaging a wider range of stakeholder in the whole COS development process, all which require funding.

Endorsement of COS by professional associations or by funders and regulatory agencies was not viewed as a major enabler for use of a COS cited by LMIC stakeholders despite it being highlighted by HIC researchers as a potential enabler [22]. Since knowledge of COS is generally lower in LMICs, it is possible that the institutions in these settings are also not yet familiar with COS and as such have not considered endorsement. This perhaps is an opportunity also to undertake sensitization and dissemination of relevant COS to these institutions which may then lead to more sustained COS use in LMICs.

### Study limitations

This survey provides insights on perceived barriers and facilitators to use of COS in LMICs and suggests some solutions. The findings of this study should be interpreted considering the following limitations. Firstly, even though there maybe variations amongst LMICs particularly between low income, lower-middle and upper middle-income countries, often referred to as low and middle income countries, the number of respondents from upper middle income countries was low, and those who had had experience with COS was even lower making it impossible to provide findings by this stratification. It is possible that even though we used TGHN, which has a huge pool of researchers, the second round of invites was even more targeted to the methodology hub within TGHN and we may have missed groups that are not represented in the network However, since our aim was to explore the experiences of various stakeholders, Despite having only 89 respondents, even though the survey information sheet was visited over 2500 times, and we achieved our aim as evidenced by the recurrence of themes. One of the reason for low response rate could be due to the timing (December/January/February) when most are usually on holiday.

Secondly, despite piloting the survey, some of the participants may have mis-interpreted some questions and as such we had a few responses that were not relevant to the question asked. This was the case for three respondents and is unlikely to alter the general findings. Finally, the survey was conducted in English, limiting the number of possible responses from French and Portuguese speaking countries. There were two responses that were in non-English language, but we feel that this is a small number to alter the findings.

## Conclusion

This survey has shown the need to raise awareness and train LMIC stakeholders not only on the utility of COS but also on the COS that are available and how to access them in locally understandable languages. Collaborations between COS developers and users of COS could provide an entry point for awareness raising and training. Feasibility of outcome measurement in LMIC settings has to be thought through as the COS is developed. Future work could assess the feasibility of using a COS developed for a HIC in an LMIC not only for research but also in informing routine data collection.

## Supporting information

S1 File Survey Questions for LMIC stakeholders. (PDF)

S2 File Country of all survey respondents. (PDF)

S3 File Responses on the case scenarios by those familiar with COS. (PDF)

S4 File Suggestions to improve wider LMIC stakeholders’ engagement in COS. (PDF)
