# Supplementary material for: Awareness and experiences on core outcome set development and use amongst stakeholders from low- and middle- income countries: An online survey
Source: PLOS Glob Public Health. 2023 Dec 5;3(12):e0002574. doi: 10.1371/journal.pgph.0002574 (PMC10697587; doi:10.1371/journal.pgph.0002574)
Supplement: S1 File — (PDF) [file pgph.0002574.s001.pdf]

Exploring the levels of awareness and experiences of stakeholders from low- and middle- income countries in core outcome set development and use.

Demographics and basic information

1. Which of the following categories best describes your current role? (Required)
   a. Clinical disciplines
      i. Public Health
      ii. Infectious diseases
      iii. General medicine
      iv. Paediatrics/child health
      v. General surgery
      vi. Nursing
      vii. Obstertrics
      viii. Gynaecology
      ix. Orthopaedics and trauma
      x. Anaesthesia
      xi. Cardiology
      xii. Haematology/Blood disorders
      xiii. Oncology
      xiv. Rheumatology
      xv. Pharmacy
      xvi. Dentistry and oral Health
      xvii. Ear, nose & throat
      xviii. Endocrine & metabolic
      xix. Eyes & vision
      xx. Psychiatry/mental health
      xxi. None
      xxii. Other (Specify)
   b. Research methods
      i. Trials management
      ii. Data management
      iii. Statistics
      iv. Ethics
      v. Information and Communication system
      vi. Effective practice/health systems
      vii. Epidemiology
      viii. None
      ix. Other (Specify)
   c. Laboratory science
      i. Biomedical sciences
      ii. Biochemistry
      iii. Microbiology
      iv. Biotechnology/genetics
      v. Other (Specify)
   d. Other disciplines
      i. Social Sciences
      ii. Health management/administration
      iii. Provider of research training
      iv. Journal /publishing staff

v. Health care assistant / or other hospital support staff
vi. Student
vii. Other (specify)

2. What type of establishment do you primarily work for/study at? (required)
    a. Academia (university, college….,)
    b. Commercial Research Organisation
    c. Community Health Centre/Facility
    d. Consultancy
    e. Government Ministry
    f. Government research organisation
    g. Hospital (Private)
    h. Hospital (Public)
    i. Industry (including Pharma)
    j. International organisation (IGO)
    k. Journal / Publishing company
    l. Non-government organisation (NGO)
    m. Public Health institute
    n. Regulatory organisation
    o. Self-employed
    p. Unemployed
    q. Other

3. For how long have you been involved in your main professional role …………….. (Time in years range from 0 to 100)
4. Which country do you work in? (drop down option) (required)
5. What is your gender?
    a. Male
    b. Female
    c. Non-binary
    d. Other (please specify)
    e. Prefer not to answer

Core outcome set questions.

1. Are you familiar with core outcome sets (COS) in research and/or clinical practice?
    a. Yes (go to question 2)
    b. No (go to question 19)

2. If yes, how did you become familiar with COS (tick all that apply)
    a. I have used a core outcome set(s) in my professional capacity.
    b. I have initiated the development of a core outcome set(s)
    c. I have been part of a team developing a core outcome set
    d. I have read a core outcome set reported in a trial.
    e. I have read a core outcome set reported/discussed in another type of research (e.g., evidence synthesis, core outcome set development paper)

   f. I have attended a conference presentation/seminar/talk on core outcome set
   g. A professional colleague/association has told me about core outcome sets
   h. Other, please specify.

3. You indicated that you have been using COS in your routine work. How did you find out about the COS that you included in your work? (For those who answered yes to 2a) (Please tick all that apply).
   a. Search of the COMET Initiative database
   b. Search of the literature
   c. Involved in previous trial that used a COS to inform outcome choice
   d. Other – please specify
4. What was your experience in using COS in your work? (Please give examples of things that went well and areas that were challenging) (For those who answered yes to 2a) [open ended response]

5. What motivated you to take part in the COS that you were involved in? (For those who answered yes in 2b and 2c) (tick all that apply) (*Required)*
   a. Prevalence of the disease in my country
   b. Prevalence of the disease/condition in low-and middle-income countries (LMICs)
   c. Working collaborations with high income countries (HICs) stakeholders who were undertaking the COS work
   d. Personal clinical/research interest in the condition
   e. Other (Specify)

6. At what stage were you involved in the development of a core outcome set? (for those who answered yes in 2b and 2c) (Tick the stages of COS development that you were involved in) Tick all that apply
   a. Determining the scope of the COS, i.e., as part of the research team
   b. Development of the protocol for the development of the COS – the 'what' to measure, i.e., as part of the research team
   c. Determining 'what to measure', i.e., giving their views on what to measure part of the consensus building process
   d. Determination of 'how to measure' the COS, i.e., giving their views on how to measure
7. For what area was the core outcome that you were involved in, developed? (for those who answered yes in 2b and 2c). Tick all that apply
   a. Anaesthesia and pain control
   b. Blood disorders
   c. Cancer
   d. Child health
   e. Consumer & communication strategies
   f. Dentistry & oral health
   g. Developmental, psychosocial & learning problems
   h. Ear, nose & throat
   i. Effective practice/health systems
   j. Endocrine & metabolic
   k. Eyes & vision

l. Gastroenterology
m. Genetic disorders
n. Gynaecology
o. Health care of older people
p. Heart & circulation
q. Infectious disease
r. Kidney disease
s. Lungs and airways
t. Mental health
u. Methodological & diagnostic
v. Muscle disease
w. Neonatal care
x. Neurology
y. Orthopaedics & trauma
z. Pregnancy & childbirth
aa. Public Health
bb. Radiology
cc. Rehabilitation
dd. Rheumatology
ee. Skin
ff. Tobacco, drugs & alcohol dependence
gg. Urology
hh. Wounds
ii. Other

8. Have you had any training in the development of COS? (For those who answered yes in of 2b and 2c)
    a. Yes
    b. No

    If yes, from where did you get this training?
    i. As part of general research methods training
    ii. As part of conferences/webinars organized by The Global Health Network/COMET Initiative
    iii. Continuous medical education session through my professional association.
    iv. Other …………..(specify)

9. Have you had any sensitization on the use of COS? (For those who answered yes in any of 2a, 2b, 2c)
    a. Yes
    b. No

    If yes, from where did you get this sensitization?
    i. As part of general research methods training
    ii. As part of conferences/webinars organized by The Global Health Network/COMET Initiative
    iii. Continuous medical education session through my professional association.
    iv. Other …………..(specify)

Scenario 1

Please read the following case scenario and answer the question that follows.

One of the leading causes of maternal mortality and morbidity in low- and middle-income countries is untreated pre-eclampsia. Pre-eclampsia is a condition experienced by some women during pregnancy and manifests as high blood pressure and protein in the urine. This condition can lead to serious complications for the mother and her baby due to the mother's placenta not functioning properly thereby, limiting the blood supply to the unborn baby.

Research on effective and safe treatment are being undertaken across various settings. The need for harmonized outcomes selection, measurement and reporting has led to development a standard set of maternal and offspring outcomes.

The list of potential core outcomes was generated through literature review (79 trials) and 30 in-depth interviews of women with lived experience followed by a three round Delphi online survey. (In a Delphi survey, results from a previous round are presented and re-scoring is undertaken after reflection) The online survey participants were healthcare professionals, researchers and women with lived experience of pre-eclampsia (281 healthcare professionals, 41 researchers and 110 women with lived experience of pre-eclampsia), from 56 countries (31 high income countries and 25 low- and middle-income countries). A consensus meeting was then held to finalise the COS, using a nominal group technique (a structured method for group discussion where members are divided into small teams, with each team feeding back to the whole group) with 16 participants (researchers, clinicians and women) resident in UK who had completed the Delphi. All the work was conducted in English.

Fourteen maternal core outcomes (death, eclampsia, stroke, cortical blindness, retinal detachment, pulmonary oedema, acute kidney injury, liver haematoma or rupture, abruption, postpartum haemorrhage, raised liver enzymes, low platelets, admission to intensive care required, and intubation and ventilation) and eight offspring core outcomes (stillbirth, gestational age at delivery, birthweight, small-for-gestational-age, neonatal mortality, seizures, admission to neonatal unit required and respiratory support) were agreed upon as the minimum dataset that should be reported in all research in pre-eclampsia.

10. Hypothetically, if you were engaged in pre-eclampsia research, based on the above case scenario, would you consider using the COS developed through the above-described process in your research?
    a. Yes
    b. No
    c. Not sure.
    Please explain your response.

Scenario 2

Please read the following case scenario and answer the question that follows.

The outcomes reported in trials in coronavirus disease 2019 (COVID-19) are extremely heterogeneous and of uncertain patient relevance, limiting their applicability for clinical decision-making. An international project was undertaken to develop a core outcome set for studies in people with suspected or confirmed COVID-19

An initial proposed list of 25 outcomes was generated by a Steering Committee, which included patients and members of the public through a review of the literature on published trials and core outcome sets. This list was subjected to an online survey involving 9,289 respondents (776 people with COVID-19 or family members, 4,882 health professionals and 3,632 members of the public) from 111 countries (48 high income countries and 63 low- and middle-income countries). The survey was conducted in five languages (English, Chinese, Italian, Portuguese, and Spanish).

Four internationals online multistakeholder consensus workshops were then convened, conducted in English in a similar manner but at different times over two days to accommodate different time zones. Participants were presented with the top 10 rated outcomes from the survey. There was a total of 95 workshop participants (people with suspected/confirmed COVID-19, members of the general public, clinicians, health researchers, funders and policy makers) for the 4 workshops from 21 countries (19 high income countries and 2 low- and middle-income countries)

Mortality, respiratory failure, multiple organ failure, shortness of breath, and recovery are critically important outcomes to be consistently reported in COVID-19 trials.

11. Hypothetically, if you were engaged in COVID-19 research, based on the above case scenario, would you consider using the COS developed through the above-described process in your research?
    a. Yes
    b. No
    c. Not sure.
       Please explain your response.

Scenario 3

Please read the following case scenario and answer the question that follows.

Palliative care is a particular way of caring for people who have diseases that cannot be cured. The main aims of palliative care are to reduce pain and to maintain the best possible quality of life as death approaches. Palliative care is commonly practiced for people with cancer and other diseases that can not be cured like dementia. When capturing patient-level outcomes in palliative care, it is essential to identify which

outcome domains are most important and focus efforts to capture these, to improve quality of care and minimise collection burden.

An international expert consensus workshop using nominal group technique (a structured method for group discussion where members are divided into small teams, with each team feeding back to the whole group) was convened within the 9th World Research Congress of the European Association for Palliative Care in 2016. Participants were identified through Outcomes
Assessment and Complexity Collaborative and Palliative Care Outcome Collaboration networks, through authorship of the European White Paper on Outcome Measurement, and through screening of oral presentation titles for the 9th World Research Congress of the EAPC 2016. All participants had to be working in palliative care (research and/or clinical) and actively using outcome measures or scientific publications about outcome measures. The workshop was a closed session lasting approximately 90 min with participants being divided into groups of 6 to 10 people to optimise discussion.

There were 33 (clinicians and researchers) from 11 countries (10 high income countries and 1 low-middle income country).

The domains of 'overall wellbeing/quality of life', 'pain', and 'information needs/preferences' are recommended for regular measurement.

12. Hypothetically, if you were engaged in palliative care research, based on the above case scenario, would you consider using the COS developed through the above-described process in your research?
    a. Yes
    b. No
    c. Not sure.
    Please explain your response.

13. How would you rate the following motivators of your using a particular COS in your professional work? For each of the motivators please rate as: 1-3 (least important), 4-6 (important), 7-9 (most important).

S.No	Question	Importance Rating
1	Knowledge of utility of COS in the wider research and clinical practice	
2	Outcomes in a COS will be more patient centered due to wide stakeholder involvement	
3	Availability of COS guidelines and resources	
4	Having stakeholders from similar settings to mine (geographical and/or resource setting) being involved in the COS development	
5	Availability of validated tools to measure the outcomes that have been proposed in the given COS	
6	Endorsement by my professional network/association	

7	Recommendations by funders or regulatory agencies in my country of work	

14. How would you rate the following barriers (that have been identified in literature) to using COS in your research or clinical area for a COS that is already available? For each of the barrier please rate as: 1-3 (least important), 4-6 (important), 7-9 (most important).

S.No	Question	Importance Rating
1	Lack of knowledge about existence of COS in my area of work	
2	The need for me to use my own outcomes or locally contextualized outcomes	
3	COS would limit the range of outcomes I would like to assess or track	
4	Lack of skills on how to apply COS	
5	Too wide or narrow scope of COS which have already been developed	
6	Inapplicability of COS developed in other geographical and resource settings to my own setting	
7	Lack of validated tools to measure the outcomes that are outlined in the COS	
8	Costly methods of measuring the outcomes in the COS	
9	Increasing burden to the clinicians and patients when additional outcomes (COS) need to be reported	
10	Outdated COS in my line of work	
11	Outdated COS measurement methods	
12	Lack of COS material in my local language	

15. Would you be willing to be part of a COS development process if asked to participate? (for those who answered yes in any of 2a, 2d, 2e, 2f, 2g)
    a. Yes
    b. No

    If yes, what would motivate you to be part of a COS development team? (open ended question)

16. In your opinion, what should be done to enable wider stakeholder engagement in development of COS in LMICs? (open ended question)
17. Do you think translation of surveys and other Delphi materials into locally used language (other than English) would increase stakeholder engagement in COS development?
    a. Yes
    b. No
    c. Not sure

    Please explain your response

If Yes, for which group of stakeholders, would translation be most useful? ……(open ended)

18. For any COS developed, there needs to be a consideration of how it will be used. This could be through having an implementation plan for the COS which would include the role of various stakeholders in the COS implementation.
    In your opinion, what should be done to enable wider stakeholder engagement in implementation of COS in LMICs? (open ended question)

19. Do you have any final comments that you would wish to make on the topic of core outcome sets? (open ended question)

The following set of questions are for those who are not familiar with COS

Core Outcome Set Description

A core outcome set (COS) is an agreed standardised set of outcomes that should be measured and reported as a minimum in all clinical trials in a specific area of health or healthcare. COS are intended to increase standardisation of outcome measurement and reporting to better enable comparisons between, and synthesis of findings of trials in a particular health area. As COS are also developed using consensus processes with key stakeholders, they also maximise the likelihood that outcomes reflect priorities of decision-makers, including patients.

After reading the above description of what COS is, please answer the following questions.

1. How would you rate the following motivators of your using a particular COS in your professional work? For each of the motivators please rate as: 1-3 (least important), 4-6 (important), 7-9 (most important).

S.No	Question	Importance Rating
1	Knowledge of utility of COS in the wider research and clinical practice	
2	Outcomes in a COS will be more patient centred due to wide stakeholder involvement	
3	Availability of COS guidelines and resources	
4	Having stakeholders from similar settings to mine (geographical and/or resource setting) being involved in the COS development	
5	Availability of validated tools to measure the outcomes that have been proposed in the given COS	
6	Endorsement by my professional network/association	
7	Recommendations by funders or regulatory agencies in my country of work	

2. How would you rate the following barriers (that have been identified in literature) to using COS in your research or clinical area for a COS that is already

available? For each of the barrier please rate as: 1-3 (least important), 4-6 (important), 7-9 (most important).

S.No	Question	Importance Rating
1	Lack of knowledge about existence of COS in my area of work	
2	The need for me to use my own outcomes or locally contextualized outcomes	
3	COS would limit the range of outcomes I would like to assess or track	
4	Lack of skills on how to apply COS	
5	Too wide or narrow scope of COS which have already been developed	
6	Inapplicability of COS developed in other geographical and resource settings to my own setting	
7	Lack of validated tools to measure the outcomes that are outlined in the COS	
8	Costly methods of measuring the outcomes in the COS	
9	Increasing burden to the clinicians and patients when additional outcomes (COS) need to be reported	
10	Outdated COS in my line of work	
11	Outdated COS measurement methods	
12	Lack of COS material in my local language	

3. Would you be willing to be part of a COS development process if asked to participate?
    a. Yes
    b. No

    If yes, what would motivate you to be part of a COS development team? (open ended question)

4. For any COS developed, there needs to be a consideration of how it will be used. This could be through having and implementation plan for the COS which would include the role of various stakeholders in the COS implementation.

    In your opinion, what should be done to enable wider stakeholder engagement in implementation of COS in LMICs? (open ended question)

5. Do you have any final comments that you would wish to make on the topic of core outcome sets? (open ended question)
