# Supplementary material for: Awareness and experiences on core outcome set development and use amongst stakeholders from low- and middle- income countries: An online survey
Source: PLOS Glob Public Health. 2023 Dec 5;3(12):e0002574. doi: 10.1371/journal.pgph.0002574 (PMC10697587; doi:10.1371/journal.pgph.0002574)
Supplement: S2 File — (PDF) [file pgph.0002574.s002.pdf]

**Country of all survey respondents**

| Country of respondent | Yes | No | Total |
|---|---|---|---|
| Bangladesh | 1 | 0 | 1 |
| Belgium1 | 0 | 1 | 1 |
| Botswana | 0 | 2 | 2 |
| Brazil | 1 | 0 | 1 |
| Burkina Faso | 0 | 1 | 1 |
| Cameroon | 0 | 2 | 2 |
| Canada | 1 | 0 | 1 |
| Chile | 1 | 0 | 1 |
| Colombia | 1 | 0 | 1 |
| Congo, Democratic Republic of the | 0 | 1 | 1 |
| Ecuador | 0 | 1 | 1 |
| Ethiopia | 0 | 6 | 6 |
| Gabon | 1 | 0 | 1 |
| Ghana | 1 | 1 | 2 |
| Haiti | 0 | 1 | 1 |
| India | 4 | 3 | 7 |
| Iraq | 0 | 1 | 1 |
| Jamaica | 0 | 1 | 1 |
| Kazakhstan | 1 | 0 | 1 |
| Kenya | 4 | 5 | 9 |
| Latvia | 0 | 1 | 1 |
| Malawi | 0 | 5 | 5 |
| Mexico | 0 | 1 | 1 |
| Nigeria | 2 | 7 | 9 |
| Rwanda | 1 | 0 | 1 |
| Saudi Arabia | 0 | 1 | 1 |
| Sierra Leone | 0 | 1 | 1 |
| South Africa | 0 | 1 | 1 |
| Spain | 1 | 0 | 1 |
| Tanzania | 0 | 2 | 2 |
| The Gambia | 0 | 1 | 1 |
| Uganda | 2 | 5 | 7 |
| United Kingdom | 3 | 0 | 3 |
| Zambia | 1 | 2 | 3 |
| Zimbabwe | 0 | 2 | 2 |
| **Grand Total** | **26** | **55** | **81** |
