# Supplementary material for: Awareness and experiences on core outcome set development and use amongst stakeholders from low- and middle- income countries: An online survey
Source: PLOS Glob Public Health. 2023 Dec 5;3(12):e0002574. doi: 10.1371/journal.pgph.0002574 (PMC10697587; doi:10.1371/journal.pgph.0002574)
Supplement: S3 File — (PDF) [file pgph.0002574.s003.pdf]

Responses on the case scenarios

S.No	Country	Pre-eclampsia	10.a. Please explain your response	COVID-19	11.a. Please explain your response	Palliative care	12.a. Please explain your response	Identified theme/s. (General or specific)
R1	Uganda	Yes	I will issue a referral from to professional specialist concerning the case	Yes	Suspend the ongoing active and put this person in isolation rooms under treatment, and be in quarantine	Not sure	Because that's so much risk the situation is between live and death	General (None)
R2	India	Not sure	..	Yes	..	Yes	..	None
R3	Nigeria	Yes	COS, is a guide on any research setting to be followed as a standard and principles.	Yes	Respect for the participants and consent is very important.	Yes	Respect for the participants, the UN convention on the human rights to social, justice and health.	General (Adherence to research guidance)
R4	Gabon	Yes	To make sure that my results could be comparable to other studies	Yes	To facilitate the data comparison and synthesis	Yes	Facilitate data comparison	General (Enhance comparability)
R5	Ghana	Yes	the COS would serve as the basis and standardized mold for guiding the researchers.	Yes	the COS would serve as the basis and standardized mold for guiding the researchers.	Yes	the COS would serve as the basis and standardized mold for guiding the researchers.	General (Adherence to research guidance)
R6	United Kingdom	Yes	I'm sure there are some and need to be able to compare with another research	Not sure	No specific question	Not sure	Not sure about qualitative measures	General (Enhance comparability)
R7	Spain	Not sure	Other aspects (feasibility of data collection, etc.) should be assessed.	Not sure	Idem	Not sure	Idem	Specific - Feasibility of outcomes measurement
R8	India	Yes	Yes I will	Yes	Yes I will	Yes	Yes I will	General (None)

R9	India	Yes	If the outcomes are feasible, these can be used.	Yes	If the outcomes mentioned are relevant to the site where the research is being conducted, can be considered	No	These COS may not be relevant to me because the responses were mostly from High income countries	Specific<br>Feasibility of outcomes measurement<br>Relevance to my setting
R10	Kazakhstan	Yes	For early detection of preeclampsia, I would develop a questionnaire with the ability to quickly identify criteria for severity and make a decision	Yes	In the clinic where I work as a consultant, alarming indicators were identified in the condition of patients with COVID-19, when it was necessary to urgently make one or another decision in changing approaches to pharmacotherapy.	Yes	For quality care in palliative patients, it is important to assess the physical, mental, emotional state of the patient, assess his pain status. Based on this, the type and volume of palliative care is built	Specific - Enhance patient centered care
R11	Rwanda	Yes	it is necessary to use cos developed for good follow up the patients and find good results.	Yes	yes, i consider using the Cos developed through the above-described process in my research for clarifying exactly which is predominant.	Yes	In this palliative care research, I consider using the COS developed through the above-described process in my research because it can show me the way to get more information needed for wellbeing, quality of life, and to reduce the pain until the end patients.	Specific - Enhance patient centered care
R12	Colombia	Yes	Because it includes the most prevalent and potential causes of death or severe disability in women and children derived from pre-eclampsia in general population	Not sure	I would consider to include economic, job, education and daily life impact of COVID-19 diagnosis or suspected infection. Despite being only 2 low- an middle-income countries in the study, the wide range of variability in social and cultural environments from these countries could allow a better understanding regarding the role of COVID-19 in health of people	Yes	the outcomes measured are appropriate. However, socio-economic field is not representative to make decisions with a global impact	Specific<br>1. Comprehensiveness of COS<br>2. Extent and range of stakeholder engagement
R13	United Kingdom	Yes	these are universal standards which have involved multiple	Yes	I would feel reassured this has taken the consideration of multiple countries and it appears	No	Insufficient LMIC engagement this only represents attendees who could afford to attend	Specific<br>1. Extent and range of stakeholder engagement

			countries leading to the core outcomes. Although the final Delphi has only UK participants and the women who participated needed to speak English, this is at least an agreed point to start from.		multiple opportunities have been provided to engage in the process. However, it could be argued the lack of LMIC participants at the workshops because technology did not enable them attend and a face to face meeting would have provided a better turn out. However as above, it is a starting place.		and numbers of participant is low.	2. Language barrier
R14	Kenya	Yes	I will consider it to try my best with my skills to reduce if not eliminate the maternal child complications and mortality which could easily be controlled if identified early.	Yes	COVID-19 being an alarming pandemic, it's good to be part of the researching group to put my effort and find solution	Yes	Cancer being one global deadliest condition with no cure at end stages. I would like to be part of the team searching the best way to handle the patients and give them hope no matter their situation.	Specific<br>Enhance comparability
R15	Kenya	Yes	The COs will help in managing the conditions and even preventive measures during antenatal visits. Regular monitoring should will be emphasized and using the current guidelines for management of pre-eclampsia. Diet should also be given priority.	Yes	Overall, infection from one person is a threat to the whole community, however emphasis should be on key messages that will help to cup the spread I would consider preventive measures from the two classes.	Yes	The main aim here is to relief the pain and give message of hope. The COS is to see the patients responding well and pain relieved.	Specific<br>(Enhance patient centered care)

R16	Kenya	Not sure	I have not had experience developing a core outcome set and therefore I am not sure if the population used would be adequate. Also, the Delphi survey was only done in the UK so I am not sure if the response in a developing country would reflect the same outcome choices	No	Why only 2 LMICs included in the workshops?	Not sure	I think the pooling of information from experts is good but I am not sure if patient representation and LMIC representation is enough	Specific<br>1. Relevance to my setting<br>2. Extent and range of stakeholder engagement
R17	Uganda	Not sure	Outcomes like; kidney injuries, stroke, seizure, liver hematomas don't manifest so much only in maternal but in other chronic disease hence hard to review specific causes.	Yes	The outcomes reported are all associated to covid 19 infection hence uniformity and acceptability to it use.	Not sure	I may to some extent consider COS but with reservation especially in low-income countries because generalization population conforms to high income country hence bias. 50:50 much suited for generalization.	Specific<br>1. Relevance to my setting<br>2. Comprehensives of the COS
R18	Canada	Yes	Systematic review of evidence, Delphi and assuming a transparent consensus process, would meet criteria for systematic identification of COS. I would note the English-only limitation for representativeness.	Yes	I would consider it, if it is the best available, but would also look for added COS in this field with more transparency in the process from evidence to consensus	Not sure	Depends. I am unsure whether this is the best available COS effort, or whether others already exist, with greater representation across LMIC. Unsure whether evidence was systematically consulted as a foundation for this.	Specific<br>1. Language barrier<br>2. Extent and range of stakeholder engagement

R19	India	Yes	Have participated in a study with WHO where similar outcomes were used	Yes	This covers the major critical events with Covid hence valid to be used as Core outcomes	Not sure	Sleep problems bowel control are also major issues. Would consider adding them	Specific<br>1. Comprehensiveness of the COS<br>2. Adherence to guidelines
R20	Chile	Not sure	22 COS in total, with no hierarchy or grouping according to objectives or research phases, seems too much to me, and may bring confusion	Not sure	I certainly prefer the COS proposed by WHO that considers the different severity of Covid-19 clinical presentations	Yes	I agree with the procedure undertaken and the selection of expert researchers as participants of the working groups	Specific<br>1. Feasibility of outcomes measurement<br>2. Extent and range of stakeholder engagement
R21	Bangladesh	Yes	Because COS will help us to identify the main complications of Pre-eclampsia.	Yes	Because it's help me quickly to take any decision about Covid-19 Cases.	Yes	I will do it. Because It's necessary to take any action by COS methods working effectively.	Specific<br>Enhance patient centered care
R22	Kenya	No	All researchers leading the team are UK residents meaning they might only have a developed country view of the study. And being the technical team, it will automatically influence the end results which may not reflect the real outcome because they might ignore some key variables or timing or procedure that might have been added by someone who live in LMIC. Second using English as the only language will jeopardize results because some	Yes	The inclusion of review team is OK. The languages used are OK	No	I think LMIC is most affected due to availability of resources so it could have been better to have more individuals from LMIC to understand how best they think the care can be handled	Specific<br>1. Relevance to my setting<br>2. Extent and range of stakeholder engagement<br>3. Language barrier

			understand of the English wording may be different. Or illiterate individual will be left out					
R23	Zambia	Not sure	Liver enzymes, renal function and platelets not easily measured in my setting. Expertise to identify retinal detachment not available. Expertise to recognize cortical blindness limited. Neonatal seizures would only be recognized if clinical. Neonatal EEG not routinely available. Intubation not available at all study sites.	Not sure	multiple organ failure needs to be more specific as to what is included and how it would be ascertained. Ability to make this diagnosis in my setting may be limited given limited diagnostic resources. Respiratory failure needs to be operationalized to assure people dying of stroke, etc. are not inadvertently included. Ideally, measures of long covid would be captured if study permitted.	No	Without further details on how these characteristics should be assessed and the role of proxy's here, this seems pretty nebulous.	Specific<br>1. Feasibility of outcome measurement<br>2. Relevance to my setting
R24	United Kingdom	Yes	A consensus of opinion on what should be reported on may give greater insight into the issue than a disparity of results. The resultant outcomes would then enable support/treatment to be harmonized/ more focused. It may also identify other trends that are not being highlighted	No	Concern over cultural variations and understanding of the key outcomes within the participant groups. The use of a single language may impact on level of understanding in regards to terminology et c The chosen suspected /confirmed COVID diagnosis is too broad.	Not sure	These may be good markers to ensure that services are targeted appropriately but service user and carers could have been involved in this	Specific<br>1. Enhance comparability<br>2. Relevance to my setting<br>3. Language barrier<br>4. Extent and range of stakeholder engagement<br>5. Feasibility of outcome measurement

R25	Nigeria	Yes	Because the process through which the COS were developed involved almost all the necessary stakeholders on the topic or issue. Though, its limitation is that it was only conducted in English language	Yes	The COS were also generated through involvement of all the necessary stakeholders. Additionally, the process use five languages and more diverse stakeholders. Hence, it's more detailed or thorough that the first process	Not sure	The process through which the COS were developed did not involve an important stakeholder in palliative care (patients receiving or who had received the care	Specific<br>1. Extent and range of stakeholder engagement<br>2. Language barrier
R26	Brazil	No	Based on the data obtained from literature review (offer the medicine based in evidence) added am interviews (with different point o view of any actors who lived or work or search about pre eclampsia problem) and after the discussion using a rigorous method to find the similarities and divergences, the core emerged from this process will be reliable but is related a specific place and we could be generalize it.	Yes	because the data are from different countries and languages, what reduce de bias and the literature evidence ws from clinical trials. In addition, the sample was considerable good number, in my opinion, the patient sample could be larger.	No	it is a consensus only from a small group from different countries, in my point of view it is not reliable and could not generalized	Specific<br>1. Relevance to my setting<br>2. Extent and range of stakeholder engagement
