# Supplementary material for: Awareness and experiences on core outcome set development and use amongst stakeholders from low- and middle- income countries: An online survey
Source: PLOS Glob Public Health. 2023 Dec 5;3(12):e0002574. doi: 10.1371/journal.pgph.0002574 (PMC10697587; doi:10.1371/journal.pgph.0002574)
Supplement: S4 File — (PDF) [file pgph.0002574.s004.pdf]

Suggestions to improve LMIC stakeholders' engagement in COS

Number	Country	Reason provided by participant	Ways of enhancing LMIC engagement. (Identified themes)
R1	India	Rich discussions, knowledge sharing	None
R2	India	All good .	None
R3	Nigeria	The application of partnership and collaboration principles	Enhancing partnerships and collaboration
R4	Kazakhstan	Simple indicators needed	Enhance feasibility of measurement
R5	Rwanda	Experienced persons and my colleagues	None
R6	Gabon	Involve more stakeholders from LMICs	Wide range and number of stakeholders
R7	Colombia	To increase stakeholder participation, at least in Latin America, calls can be opened at the community level (community leaders, public universities, government programs) offering educational and work incentives to stakeholder representatives involved in a given study. This would stimulate committed participation with a high level of quality, always pursuing the interests of potential beneficiaries.	Educational and work incentives to participate and use COS
R8	UK	Different languages and access to discussions. Global webinars are good we hear all views but when there is a lack of engagement consider other methods. It may also be more appropriate to set a standard for a HIC and LIC setting separately as they have different priorities. Given different levels of access to health, living conditions and global environmental impacts. However, we need to learn from one another about what is important in different settings identify what is consider a good outcome for the individuals. Identify key core elements of health outcomes i.e. what constitutes an improvement or benefit to an individual in one setting may not be the same in another.	Use of online platforms to enable wider participation<br><br>Translation into local language<br><br>Relevance of COS to a given setting<br><br>Partnership and collaboration
R9	Kenya	Increase on educating more medical workers on the use of cos	Education on COS
R10	Ghana	Directly engaging the clinicians at the primary health care level where those clinicians are community-based. Engaging both clinicians and the community at the local level directly would ensure representativeness instead of always engaging the clinicians at the tertiary level of care. The clinicians at the primary health care level	Sensitization on COS at different levels<br><br>Enhance public participation<br><br>Dissemination of COS and measurement tools

		should be empowered with the necessary tools and methods relevant to COS.	
R11	Kenya	Partner Involvement	Enhancing partnerships and collaboration
R12	Uganda	Developing more COSs	Enhance availability of COS
R13	Kenya	Requesting for feedback from healthcare professionals in practice wherever possible and giving them a chance to collect the opinions of the patients they service will increase interest and build capacity	Monitoring and evaluation of COS implementation<br><br>Audit and feedback by COS users
R14	Uganda	Involvement of institutions especially universities LMICs during the early phase and training of man powers in these marginalised territories.	Enhancing partnerships and collaboration<br><br>Education on COS
R15	Canada	Improved outreach to inform about opportunities to contribute to COS (and the importance and relevance of doing so). Improved representation of stakeholders (beyond clinicians) in LMICs. Opportunity to understand how engagement contributes to better research and care for different stakeholder considerations (i.e. opportunity to 'follow' the COS development and implementation that they have contributed to). Opportunity to be involved in follow-up for future iterations.	Sensitization on COS<br><br>Enhance public participation<br><br>Monitoring and evaluation of COS implementation
R16	India	Hardly anyone in my circle has heard of COS. The first step would be to disseminate knowledge on what COS is and what are its benfits followed by training sessions -online or offline	Sensitization on COS<br><br>Training on COS
R17	Chile	Sorry, haven't thing about it	None
R18	UK	Engage with The James Lind Alliance	Enhancing partnerships and collaboration
R19	Bangladesh	Concern of awareness to engage them.	Sensitization on COS
R20	Kenya	Proper communication	Enhancing partnerships and collaboration
R21	Brazil	establishing a good connection with them creating a network to involve them maintaining often communication with the showing we are interested in their practical reality	Enhancing partnerships and collaboration
R22	Zambia	More involvement from researchers and clinicians in LMIC settings.	Engagement of wider range of stakeholders
R23	Spain	Better communicate the importance of COS in research Include it as a requirement in calls for funding.	Sensitization on COS

			Embed it as a funders requirement
R24	UK	Teaching in curriculum	Education on COS
R25	Nigeria	(1) Increased awareness about the existence of COS and the need to use it . This can be done through engagement with various professional bodies and ministries of health in these regions (2) Involvement of governments in the regions, to "buy into" the need for COS , with a view to ensuring political will and policies to support development of COS for treatment of each clinical condition,and enusre it's used. (3) Research funders should also recommend the use of COS for any clinical trial they will be sponsoring in the regions (4) Regular training of researchers on COS (5) International or foreign researchers (for high-income countries) should recomend/suggest the need to have and use COS to their collaborators in LMICs	Sensitization on COS<br><br>Enhance partnerships and collaborations<br><br>Training on COS<br><br>Embed it as a funder's requirement
R26	India	It need to be made more available to people they should be more awaited of this process.	Enhance access to COS
R27	Cameroon	Il faudrait faire tout d'abord une sensibilisation sur l'importance de COS surtout auprès des patients qui participent à l'enquête	None
R28	Ethiopia	To enable wider stakeholder engagement in implementation of COS in LMICs detail awareness.	Sensitization on COS
R29	India	This paper provides a short conceptualisation of stakeholder engagement, followed by 'design principles' that we put forward based on a combination of existing literature and new empirical insights from our recently completed longitudinal study of stakeholder engagement. The design principles	None
R30	Latvia	I have not thought about that	None
R31	Iraq	N/A	None
R32	Uganda	By involving the stakeholders in the process of preparing the COS so that they get to understand it's importance and demands	Enhancing partnerships and collaboration throughout the COS development process
R33	Jamaica	Strategies that will be used to inform different stakeholder group about the benefits of COS.	Sensitization on COS usefulness at different levels.
R34	Ethiopia	We academic and clinical researcher in LMIC country really need training opportunity as well as research grant opportunity. To be fully engaged in implementation of COS in ethiopia we really need collaboration with Liverpool University and other to to be mentored.	Enhancing partnerships and collaboration<br><br>Training on COS

R35	Congo, DR	Rigueur dans la pratique	None
R36	Sierra Leone	Now the COS is a general procedure that one should acquire for any clinical trial, so it will be really important to know more of COS in order to do pure clinical trial.	Training on COS
R37	Kenya	Involving stakeholders in making COS	Enhancing partnerships and collaboration throughout the COS development process
R38	Nigeria	I don't know what LMICs mean	None
R39	Tanzania	More inputs in under developing and developing countries concerning the health care	None
R40	India	Selection and involvement of wider researcher community in the field from the area and to use their expertise to customise according to local needs without compromising global standards.	Engagement of a wider range of stakeholders<br>Enhancing partnerships and collaboration<br>Relevance of COS to a given setting by contextualization
R41	Malawi	By provide them with civic education about cos	Sensitization on COS
R42	Nigeria	I think information is very Paramount in everything, creating more awareness is the key.	Sensitization on COS
R43	Ethiopia	all stakeholders should be followed to use such criteria	Support COS implementation?
R44	Nigeria	Advocacy and sensitization, involvement of the stakeholder while developing the COS. Also, contextualizing the COS and having it in indigenous languages is imperative. I have taken the WHO Mass Online Open Course in Implementation Research, such training can be contextualized for stakeholders and end-users of the COS to enhance acceptance, applicability and fidelity.	Sensitization on COS<br>Relevance of COS to a given setting by contextualization<br>Translation of COS to indigenous language<br>Training on COS for intended users
R45	Uganda	Adequate Sensitization Having Regional Focal Persons	Sensitization on COS<br>Having a COS 'champion'
R46	Ghana	COS needs to capture adequately participants' views. Since COS are expected to be reached through consensus, this consensus will be credible only if all relevant stakeholders have a fair chance to participate and each stakeholder should have the same chance to express their perspective and to participate in decision making	Methodology rigour of COS development process<br><br>Engagement of a wider range of stakeholders
R47	Kenya	Awareness and trainings	Sensitization on COS<br>Trainings on COS

R48	Uganda	Create awareness through health education. use online platforms to share the advantages of COS share experiences from persons that have used COS	Sensitization on COS<br>Leveraging on online platforms<br>Enhancing partnerships and collaboration
R49	Belgium	To be sure that COS won't increase the administrative burden for physicians	Ensure outcomes measurement are feasible
R50	Burkina Faso	More training and advocacy	Advocacy on COS<br>Training on COS
R51	Tanzania	1.spreading awareness on the importance of developing and following the COS in LMICs to the public level. 2. involving the LMICs members during development of COS of different studies and interventions 3.translating the COS in local languages to increase awareness and 4. To make it a requirement to have COS for funders and research institutes to conduct studies.	Sensitization on COS<br>Enhancing partnerships and collaboration throughout the COS development process<br>Translation of COS to local language<br>Embed it as a funder's requirement
R52	Zimbabwe	There is need for training and information to increase stakeholder buy in.	Training on COS
R53	Zambia	There is need to request many more participants	Wide stakeholder engagement?
R54	Malawi	I feel first is to do mass media communication or sensitising the communities within the COS stakeholders, on what COS is and how it is supposed to be done.	Sensitization on COS usefulness and COS development
R55	Kenya	engagement of the communities where the research will be conducted	Community engagement
R56	Ethiopia	First there should be an agreement between stakeholder to use COS as a measurement of the outcome of once study and it should be filled out regularly to evaluate/measure or take corrective action of the study outcome regularly and timely.	Stakeholder engagement during consensus building<br><br>Monitoring and evaluation of COS implementation
R57	Botswana	Creation of COS awareness is the most critical element in this instance, awareness should be created worldwide to the targeted stakeholders so that they acknowledge and understand these concepts, for smooth acceptance/adoption and implementation withing their respective organizations in LMICs. Routine monitoring and evaluation measures should also put in place for smooth implementation in general.	Sensitization on COS<br><br>Monitoring and evaluation of COS implementation
R58	South Africa	Benefits of COS explained	Sensitization on COS utility
R59	Kenya	sensitization through education	Education on COS

R60	Saudi Arabia	Educating the enterprises on the importance of implementing COS in LMICs.	Education on COS
R61	The Gambia	Improved publicity as most people might not be aware of the concept of COS	Sensitization on COS
R62	Haiti	First of all. It's important to promote COS by share them the benefit to implemented. It's important to show it's not a waste time for the system. Using evidence base to show the experience made with another system and the improvement result from the implementation of COS.	Sensitization on COS<br><br>Monitoring and evaluation of COS implementation
R63	Botswana	There must be a wide capacity building on COS for clinicians on the ground, researchers, reviewers and regulatory body officers and academia and by extension students. All these are very strategic in ensuring that COS once developed it will be used in research and practice. Training is key to stakeholder engagement..	Training on COS<br><br>Wide stakeholder engagement
R64	Zimbabwe	Training and awareness - Parallel session at popular larger research conferences - virtual option	Training on COS<br><br>Leveraging on online platform
R65	Malawi	Pass	None
R66	Nigeria	There need to be healthcare and patient outcomes accreditation organizations to monitor the implementation of cos in research and practice in LAMICS.	Monitoring and evaluation of COS implementation
R67	Uganda	Adequate funding, involving as many regulatory bodies as possible	Availing funds for COS development and use.<br><br>Enhancing partnerships and collaborations with regulatory bodies
R68	Nigeria	Creating Awareness	Sensitization on COS
R69	Uganda	A fairer ground needs to be given to everyone to put plain their views and what they think is best for them to receive just quality and acceptable healthcare. Explain to the stakeholders the importance of COS	Sensitization on COS<br><br>Use of online platforms?
R70	Kenya	Not sure	None
R71	Ethiopia	I think it is important to increase awareness of stakeholders through meeting, panel discission. It need close work with them.	Sensitization on COS<br><br>Enhancing partnerships and collaborations

R72	Malawi	Introduce research question/trial to stakeholders	None
R73	Cameroon	Greater sensitization on the importance, value and application of COS in clinical research and clinical trials. Also, quality and adequate training opportunities should be made available for training health researchers and other personnel.	Sensitization on COS<br><br>Training on COS
R74	Ethiopia	Incorporating local stakeholders in Cos is very important for easing our work.	Wide local stakeholder engagement
R75	Ecuador	Create investigational teams for the development of different specialties or lines of investigation	None
R76	Nigeria	The rich in the country should invest in the process, likewise the celebrity	Availing funds for COS development and use.
R77	Malawi	More stakeholder consultation in LMICs before development	Enhancing partnerships and collaborations
R78	India	By exploring the impact of stakeholder engagement on guideline development and implementation.	Monitoring and evaluation of COS implementation
R79	Mexico	More information and capacitation in these areas	Training on COS
R80	Nigeria	Create more awareness.	Sensitization on COS
R81	Zambia	Training or mentorship and engagement with various stakeholders in LMICs on COS through collaborative efforts by centers of excellence could improve uptake and implementation.	Training on COS<br>Engagement of different categories of stakeholders<br>Enhancing partnerships and collaborations
